# Diversity of fungi attached to birds corresponds to the habitat ecologies of their avian dispersal vectors

**DOI:** 10.1093/aob/mcaf077

**Published:** 2025-05-03

**Authors:** Niko R Johansson, Ulla Kaasalainen, Jouko Rikkinen

**Affiliations:** Botany & Mycology Unit, Finnish Museum of Natural History LUOMUS, PL7 00014 University of Helsinki, Helsinki, Finland; Organismal and Evolutionary Biology Research Programme, Faculty of Biological and Environmental Sciences, PL65 00014 University of Helsinki, Helsinki, Finland; Botany & Mycology Unit, Finnish Museum of Natural History LUOMUS, PL7 00014 University of Helsinki, Helsinki, Finland; Botany & Mycology Unit, Finnish Museum of Natural History LUOMUS, PL7 00014 University of Helsinki, Helsinki, Finland; Organismal and Evolutionary Biology Research Programme, Faculty of Biological and Environmental Sciences, PL65 00014 University of Helsinki, Helsinki, Finland

**Keywords:** Fungi, birds, bird–fungal interactions, propagule, transport, dispersal, dispersal vector, directed dispersal, environmental DNA, habitat preference, fungal diversity, ectozoochory

## Abstract

**Background and Aims:**

Animal-mediated transport of propagules is an important aspect of dispersal in many organisms, but severely understudied in fungi. Birds appear as natural dispersal vectors for many fungi, as they are often both mobile and migratory, potentially providing directed transport of fungal propagules to suitable sites for establishment. However, information of which fungal taxa are transported by which bird vectors is lacking.

**Methods:**

By using environmental DNA (eDNA) metabarcoding of feather and feet swabs collected from bird species with two contrasting habitat ecologies (European robin *Erithacus rubecula* and goldcrest *Regulus regulus*), we identify and compare the diversity of fungi attached to these birds.

**Key Results:**

We identified over 600 fungal taxa attached to and potentially transported by the birds. Differences in the fungal assemblages of the two bird species corresponded with species-specific patterns in the natural histories of transported fungi and the bird vector.

**Conclusions:**

Our findings show that bird-mediated transport can be important for a variety of fungal taxa, especially at medium to long transport distances. Taxa particularly affected include plant pathogens, saprobic macrofungi and sorediate macrolichens, especially those with specific habitat requirements.

## INTRODUCTION

Animals are known to contribute to the dispersal of many organisms. For instance, there is a long tradition of studying bird–plant interactions and seed dispersal via frugivory (Schupp *et al*., 2010) with numerous studies showing the importance of animal-mediated dispersal at the ecosystem level (Rogers *et al.*, 2021; Mendes *et al.*, 2024). Most such studies have focused on endozoochory of vascular plants (transport of ingested fruit/seed material internally), and fewer on epi- or ectozoochory (transport of material attached to the external surface/fur/feathers of the animal). Epi- or ectozoochory may be particularly important for dispersing taxa that do not form a major part of bird diets, such as many groups of fungi (Zambonelli *et al.*, 2017; Green *et al.*, 2023; but see Elliott *et al.*, 2019). Fungi, bryophytes and algae are being increasingly investigated in the context of animal-mediated dispersal, but many of the potential dispersal interactions between fungi and animals remain unknown (Jusino *et al.*, 2016; Zambonelli *et al.*, 2017; Chmielewski and Eppley, 2019, 2022). Recognizing and describing species interactions and their ecological relevance is considered a major knowledge gap in biodiversity science, nicknamed the Eltonian shortfall (Hortal *et al.*, 2015).

For many potential fungus–animal dispersal systems, we still lack basic information even of the early stages of the dispersal process, such as which propagules can attach to which animal vectors (Vašutová *et al*., 2019; Paz *et al.*, 2021, and references therein). Direct detection and identification of fungal propagules attached to animals using microscopy is possible, but demonstrating species- or taxon-level ecological patterns is challenging (Bailey and James, 1979; Lewis *et al.*, 2014*a*; Chmielewski and Eppley, 2019; Johansson *et al*., 2021). Cultivation-based approaches can allow for more detailed species identification, but extensive surveys are difficult, labour-intensive and restricted to a limited set of cultivable taxa (Tiffany *et al.*, 1955; Evans and Prusso, 1969; Hubálek, 1978; Farris *et al*., 2004). Cultivation-based studies suggest that common fungal genera attached to birds include *Alternaria*, *Arthroderma*, *Chaetomium*, *Coniothyrium*, *Cladosporium*, *Penicillium* and *Trichoderma*, among others (see e.g. Malloch and Blackwell, 1992, and references therein). Many of these are ubiquitous, generalist saprotrophs, while others are shown to have keratinolytic activity, indicating potential residence or pathogenic/decomposer metabolic activity in the plumage (Pugh and Evans, 1970). Many bird–fungal studies have focused on dermatophytes, fungal plant pathogens relevant for agriculture and forestry, or potential human pathogens, leaving dispersal ecology patterns of many other fungal groups more or less unstudied (Heald and Studhalter, 1914; Pugh, 1965; Pugh and Evans, 1970; Hubálek, 2004).

DNA-based specimen identification and environmental DNA (eDNA) surveys have become attractive for ecologists, especially working with taxa that have traditionally been difficult to sample, detect and identify. This includes studies of bird-mediated fungal dispersal (Jusino *et al*., 2022; Shi *et al*., 2024). eDNA refers to traces of organismal DNA that can be sampled and sequenced from environmental samples, e.g. from air, soil, water or, in this case, attached to the surfaces of birds. Quantitative PCR approaches can be used for quantification of DNA amounts but typically lack detailed taxonomic resolution (Del Mar Labrador *et al.*, 2020), while eDNA allows relatively easy identification of taxa from minute quantities of biological material and is well suited to the characterization of diverse assemblages of interacting organisms. Even if eDNA metabarcoding of fungal material is conceptually straightforward, linking detected eDNA sequences to actual species or species groups remains non-trivial, rendering the interpretation of the results challenging. However, with increasing database coverage (Abarenkov *et al.*, 2023), extensive global sampling campaigns (Ovaskainen *et al*., 2020, 2024; Tedersoo *et al.*, 2021) and novel innovative sampling setups, the utility of eDNA methods is expected to rapidly increase.

Bird-mediated dispersal of fungi has been hypothesized to be prevalent at intermediate to large spatial scales (Chaudhary *et al.*, 2022) and predicted to occasionally lead to long-distance dispersal events (Lewis *et al.*, 2014*a*, *b*; Garrido-Benavent *et al.*, 2018). Some bird groups have gained considerable interest as dispersal vectors, such as waterbirds as vectors for planktonic organisms and aquatic plants (Green *et al.*, 2023), and woodpeckers as vectors for epiphytic and wood-decaying fungi (Farris *et al*., 2004; Jusino *et al*., 2016, 2022; Johansson *et al.*, 2021; Shi *et al.*, 2024). In this study, we focus on small passerine birds, which have been less explored as potential dispersal vectors for fungi. Using eDNA metabarcoding, we characterize ectozoochorous fungal assemblages of small forest birds in Finland. Besides taxonomic identification of the retrieved taxa, the transported organisms are classified to functional lifestyles whenever possible to assess the ecological relevance of bird-mediated dispersal for the detected taxa. Based on previous studies, we expect to find a diverse but non-random set of fungi attached to birds, with detectable ecological patterns between bird species, sampling positions on the bird and functional traits of the attached taxa.

## MATERIALS AND METHODS

### Field sampling

Birds were captured during the autumn migration of 2022 at the Hanko Bird Observatory in southernmost Finland (59°48′38″N, E22°53′42″E). The European robin (*Erithacus rubecula*) and the goldcrest (*Regulus regulus*) were selected for sampling based on their contrasting ecologies. In Finland, robins usually inhabit semi-urban forest patches, gardens and parklands dominated by deciduous trees. In contrast, goldcrests typically inhabit conifer-dominated forests, both in natural and in urban forested areas. Robins are considered ground passerines, as they spend a considerable amount of time on the ground, whereas goldcrests are considered foliage-gleaners and typically forage mostly in the canopy of conifer forests (Alatalo, 1981; Norberg, 2021). These different habitat preferences and foraging behaviours can be expected to be reflected in the fungal material attached to their feathers and feet. Neither of the two bird species are resident at the Hanko Bird Observatory area during the sampling period, as they are migrating towards the south.

The Hanko Bird Observatory is located on the tip of a south-facing peninsula characterized by a mosaic of habitat types typical for the Baltic sea coast, including lichen-dominated rock outcrops and boulder shores, coastal meadows dominated by grasses and forbs, shrubby thickets dominated by *Calluna vulgaris* and *Vaccinium myrtillus*, and coastal forests dominated by *Pinus sylvestris* mixed with *Betula* spp., *Populus tremula*, *Salix* spp., *Sorbus aucuparia*, *Picea abies*, *Juniperus communis* and *Alnus glutinosa*. The central, non-coastal parts of the peninsula are characterized by larger patches of dry heath forests dominated by pines and mesic herb-rich forests dominated by deciduous trees (Kontula and Raunio, 2018). A harbour area of high human activity and alteration of the landscape lies between the bird observatory and the mainland.

The birds were caught with mist-nets during standardized bird ringing and handled by permit-holding bird ringers. When caught in the net, sterile cotton swabs were applied before further handling of the bird to sample biological material attached to feathers and feet. For feather sampling, the swab was run across chest plumage in a rotational motion for 10 s. For sampling of the feet, the bird was allowed to grab the tip of the swab, followed by rotation of the swab to dislodge any biological material attached to the digits, including under the claws. The sampling was repeated using the same swab for both feet. For three individuals of each species, three repeated swabs were taken from both feathers and feet. Swabs were placed in individual sterile tubes and brought to a freezer (−18 °C) after a maximum of 1 h of field work and stored at this temperature or on ice during further transport and storage. During all field days, hourly environmental control swabs were taken during fieldwork and exposed to the ambient air for 10 s without contact with birds and handled identically in downstream sample processing and analysis. All sampling was conducted in sunny, dry weather. Altogether eight robins and ten goldcrests were sampled during two field days.

### DNA extraction, PCR and sequencing

To extract DNA, a modified protocol from Abrego *et al.* (2018) was used. The swab heads were suspended in 300 µL of sterile MQ-water, vortexed and incubated with added 75 µL of 24 % Chelex 100 resin solution (Bio-Rad, Hercules, CA, USA) and 20 µL of proteinase K (Thermo Fisher, Waltham, MA, USA) in a shaking heat block at 56 °C for 90 min to induce lysis of biological material. DNA extractions included a negative control sample (sterile swab head) per each batch of extractions. Using adapter-tailed primers ITS3 and ITS4 (White *et al.*, 1990), PCR was performed targeting the ITS2 region, a commonly used fungal (meta)barcode, using a modified protocol from Hebert *et al.* (2013). A total reaction volume of 25 µL included 12.5 µL of 10 % d(+)-trehalose dihydrate, 4 µL of sterile PCR water, 1.25 µL 50 mm MgCl_2_, 0.125 10 mm dNTP, 2.50 µL Platinum Taq buffer, 0.12 µL Platinum Taq polymerase (Thermo Fisher), 0.25 µL of 10 mm solution of both forward and reverse primers, and 4 µL of DNA template. The template was diluted 1:10 to 1:50 on a sample-by-sample basis. The PCR was run using the following conditions: initial denaturation in 94 °C for 2 min, followed by 28 cycles of denaturation at 94 °C for 1 min, annealing at 55 °C for 1 min and extension at 72 °C for 1 min. This was followed by final elongation at 72 °C for 5 min. Each PCR was run in triplicate and the product was verified using agarose gel electrophoresis. Each PCR batch included negative (MQ-water) and positive (*Agaricus bisporus* extract) controls. PCR products were sent for library preparation with dual-indexing using in-line barcodes, followed by quality checks, size selection, pooling and Illumina MiSeq paired-end sequencing by an external provider (Canadian Centre for DNA Barcoding, CCDB, Guelph, Canada). All negative controls, including environmental, DNA extraction and PCR controls, were sequenced together with the samples. The sequencing step included additional sequencing blanks (MQ-water). Altogether we sequenced 384 samples, of which 41 were various negative controls.

### Bioinformatics and statistics

Raw reads were paired and demultiplexed by CCDB. Adapters and primers were trimmed with Cutadapt (Martin, 2011) and dereplicated in OBItools (Boyer *et al.*, 2016). ITS2 sequences were extracted from these using ITSx (Bengtsson-Palme *et al.*, 2013). The frequency of tag jumps – cases where individual reads are demultiplexed into incorrect samples (Schnell *et al.*, 2015) – were estimated using non-used tag space in the sequencing step. A maximum of three reads per sequence variant were demultiplexed into this non-used tag space (data not shown). Thus, a read count threshold of four per sequence variant per sample was applied to eliminate a low level of baseline tag jumps. Clustering to operational taxonomic units (OTUs) was performed on these sequences with SUMACLUST (Mercier *et al.*, 2013) at the 97 % threshold.

For OTU annotation, the central sequence of each cluster was matched to the UNITE10 database (Abarenkov *et al.*, 2023) using the massBLASTer tool in the PlutoF platform (Abarenkov *et al.*, 2010). These matches were then manually curated, validated and, when possible, assigned to explicit species hypotheses (SHs) on the UNITE10 database. In assigning taxon names to clusters, special weight was given to Sanger-sequencing-verified voucher specimens in SHs from Finland or other nearby countries. If available, sequences from types or ex-type cultures were used to verify the taxon assignment of a given OTU. In some cases, multiple nearby OTUs were combined under the same large SH following the taxon hypothesis logic in UNITE (Kõljalg *et al.*, 2020) for downstream analysis.

For a given OTU to be recorded present in a sample, it needed to appear in at least two of the three replicate PCRs. Taxa classified as non-fungi were removed and analysed separately. OTUs with <90 % matches to any reference datasets and OTUs flagged as putative chimeras or sequencing artefacts were discarded. Negative controls included very little DNA or OTUs, but as a precaution OTUs present in these samples (nine clusters) were globally removed, apart from *Cladosporium* sp. and *Sistotrema* sp. 1. These two OTUs were present at low read counts in three and one negative controls, respectively, and were common in bird samples. These OTUs probably represent real taxa detected in birds and their presence in negative controls is probably due to (aerosolar) post-PCR contaminations of negative controls. An OTU corresponding to *Sticta sublimbata* was identified as a local laboratory contaminant and removed globally.

Each fungal OTU was assigned to one of six functional groups or lifestyles: wood-decayers, other saprotrophs, ectomycorrhizal fungi, lichen-forming fungi, plant pathogens and other pathogens/parasites. The grouping was based on the FungalTraits database (Põlme *et al.*, 2020) using the genus name as the key linking value and our own expertise. OTUs identified only to the family level, or a higher taxonomic rank, were assigned as unclassified for lifestyle.

The structure of the fungal OTU assemblages was illustrated using non-dimensional metric scaling (NDMS) with the R package vegan (Oksanen *et al.*, 2024) in the R environment (R Core Team, 2024). To test for statistical differences between control and bird samples, between bird species and between sampling positions, a non-parametric permutational multivariate ANOVA (PERMANOVA) analysis was used, again with the package vegan. Fungal assemblages were visualized with the package Metacoder (Foster *et al.*, 2017).

Produced OTUs were compared to two large global published fungal eDNA datasets: Global spore sampling project GSSP for airborne fungal eDNA (Ovaskainen *et al.*, 2020, 2024) and Global soil mycobiome consortium GSMc for soil-borne fungal eDNA (Tedersoo *et al.*, 2021), using local blast searches with rBLAST (Hashler and Nagar, 2024). A sequence similarity of ≥99 % in a length of >75 bp was considered a match between an OTU central sequence in the bird data and GSSP or GSMc data.

## RESULTS

The final dataset included 650 OTUs, of which 611 were classified as fungi, 27 as green algae and 12 as other taxa. The detected fungi encompassed taxa from at least 22 classes and all six lifestyle groups. Some examples of commonly encountered taxa are shown in Table 1. The full dataset of encountered OTUs is available in Supplementary Data Table S1. Some of the detected fungal OTUs were common globally in the dataset, but most were detected in only one or a few samples.

**Table 1.** Examples of fungal taxa commonly encountered in robins and goldcrests. For each bird species, the presence of a fungal taxon is shown as a fraction of the total number of birds sampled of that species. Fungal lifestyles and notes follow the FungalTraits database and our own expertise. The final column indicates if a 99 % (>75 bp) BLAST match of the given taxon is recovered in global sampling campaigns for aerial (GSSP; value A in the table) or soil (GSMc; value S in the table) fungal eDNA.

Functional group/lifestyle notes	Taxon	Robins		Goldcrests		Present in aerial/soil eDNA?
		Feet	Feathers	Feet	Feathers	
Litter saprotroph (hypervariable)	*Cladosporium* cf. *herbarum*	8/8	8/8	10/10	10/10	A/S
Litter saprotroph (agaricoid)	*Mycena metata*	5/8	4/8	8/10	5/10	A/S
Litter saprotroph (agaricoid)	*Mycena epipterygia s.s*.	2/8	2/8	8/10	4/10	A/S
Litter saprotroph (agaricoid)	*Mycena galopus*	2/8	2/8	7/10	4/10	A/S
Litter saprotroph (agaricoid)	*Mycena leptocephala*	1/8	1/8	6/10	3/10	A/S
Litter saprotroph (agaricoid)	*Mycena sanguinolenta*	2/8	0/8	5/10	4/10	A/S
Litter saprotroph (agaricoid)	*Mycena vulgaris*	1/8	0/8	5/10	3/10	A/S
Saprotroph (agaricoid, conifer cones)	*Baeospora myosura*	4/8	2/8	9/10	8/10	A/S
Litter saprotroph (agaricoid)	*Clitocybe metachroa/decembris*	0/8	0/8	3/10	4/10	A/S
Litter saprotroph (agaricoid)	*Clitocybe nebularis*	0/8	0/8	3/10	2/10	A/S
Litter saprotroph (agaricoid)	*Clitocybe odora* coll.	1/8	0/8	2/10	1/10	S only
Litter saprotroph (agaricoid)	*Cystoderma jasonis s.l.*	0/8	0/8	1/10	1/10	A/S
Litter saprotroph (agaricoid)	*Cantharellula umbonata*	0/8	1/8	1/10	0/10	A only
Litter saprotroph (some ectomycorrhizal, corticioid)	*Sistotrema* 1 SH1134482.09FU	5/8	3/8	6/10	5/10	A/S
Litter saprotroph (some ectomycorrhizal, corticioid)	*Sistotrema* 6 SH0895568.09FU	3/8	2/8	2/10	2/10	A/S
Litter saprotroph (some ectomycorrhizal, corticioid)	*Sistotrema sernanderi*/*oblongisporum*	2/8	0/8	2/10	0/10	A/S
Soil saprotroph (yeast)	*Vishniacozyma victoriae*/*carnescens*	3/8	2/8	1/10	1/10	A/S
Soil saprotroph (yeast)	*Vishniacozyma tephrensis*	3/8	1/8	1/10	0/10	S only
Soil saprotroph (filamentous mould)	*Mucor* cf. *circinelloides* 1	1/8	1/8	2/10	1/10	S only
Soil saprotroph (filamentous mould)	*Mucor* cf. *hiemalis*	4/8	0/8	0/10	0/10	A/S
Soil saprotroph (agaricoid)	*Entoloma turbidum*	0/8	0/8	1/10	1/10	S only
Unspecified saprotroph (filamentous mould)	*Aspergillus ruber*	8/8	5/8	5/10	3/10	A/S
Unspecified saprotroph (filamentous mould)	*Aspergillus glabripes*	6/8	4/8	5/10	3/10	S only
Unspecified saprotroph (filamentous mould)	*Penicillium* SH0939544.10FU	4/8	3/8	3/10	3/10	A/S
Unspecified saprotroph	*Perusta* cf*. inaequalis*	1/8	0/8	2/10	2/10	A/S
Wood-decaying/pathogen (conifers)	*Sydowia* cf*. polyspora*	2/8	1/8	9/10	7/10	A/S
Wood-decaying (corticioid)	*Trechispora byssinella*	3/8	1/8	2/10	1/10	Neither!
Wood-decaying (corticioid)	*Peniophora incarnata*	3/8	1/8	2/10	1/10	A/S
Wood-decaying (corticioid)	*Peniophora* cf. *junipericola*	0/8	0/8	3/10	0/10	Neither!
Wood-decaying (agaricoid)	*Hypholoma capnoides*	0/8	1/8	4/10	2/10	A/S
Wood-decaying (agaricoid)	*Hypholima fasciculare s.s*.	0/8	0/8	2/10	1/10	A/S
Wood-decaying (corticioid)	*Hyphodontia pallidula*	0/8	0/8	3/10	2/10	A/S
Wood-decaying (corticioid)	*Merulicium fusisporum*	2/8	0/8	1/10	2/10	A/S
Plant pathogen	*Didymella exigua*	4/8	3/8	3/10	4/10	S only
Plant pathogen (*Pinus sylvestris* needles)	*Lophodermium pinastri*	4/8	2/8	2/10	3/10	A/S
Plant pathogen (*Picea abies* needles)	*Lophodermium picea*	4/8	0/8	1/10	1/10	A/S
Plant pathogen (Ericaceae)	*Exobasidum* cf. *arescens*	4/8	3/8	1/10	1/10	A/S
Plant pathogen (Ericaceae?)	*Exobasidium* sp. 1 SH0895612.09FU	4/8	0/8	1/10	2/10	Neither!
Plant pathogen	*Neoascochyta europaea*	3/8	0/8	2/10	1/10	A/S
Plant pathogen (*Betula* sp.)	*Melampsoridium betulinum*	1/8	0/8	2/10	2/10	A/S
Plant pathogen (*Pinus sylvestris*)	*Heterobasidion annosum*	1/8	0/8	3/10	1/10	A/S
Plant pathogen (*Picea abies*)	*Heterobasidion parviporum*	0/8	0/8	2/10	0/10	A/S
Plant pathogen (conifer needles)	*Kabatina thujae*/*juniperi*	0/8	0/8	4/10	0/10	S only
Plant pathogen	*Iternsonilia perplexans*	3/8	4/8	1/10	1/10	A/S
Plant pathogen (foliage)	*Phyllactinia alni*/*betulae*/*fraxini*	1/8	1/8	1/10	2/10	A only
Plant pathogen	*Claviceps* cf. *purpurea*	2/8	1/8	0/10	1/10	A/S
Ectomycorrhizal (boletoid)	*Suillus bovinus*	0/8	0/8	3/10	1/10	A/S
Ectomycorrhizal (boletoid)	*Suillus luteus*	0/8	1/8	1/10	1/10	A/S
Ectomycorrhizal (agaricoid)	*Laccaria laccata*/*pumila*/*ametysthina*	0/8	0/8	0/10	2/10	A/S
Ectomycorrhizal (clavarioid)	*Thelophora terrestris*	0/8	1/8	0/10	1/10	A/S
Ectomycorrhizal (agaricoid)	*Tricholoma fulvum*	0/8	0/8	1/10	1/10	A/S
Lichen-forming (crustose)	*Scoliciosporum* cf. *umbrinum*	8/8	2/8	9/10	9/10	S only
Lichen-forming (crustose)	*Scoliciosporum chlorococcum* 1 SH0916870.10FU	3/8	2/8	1/10	1/10	A/S
Lichen-forming (crustose)	*Scoliciosporum chlorococcum* 2 SH0925046.10FU	1/8	0/8	0/10	0/10	A/S
Lichen-forming (foliose)	*Hypogymnia physodes*	5/8	1/8	7/10	4/10	A/S
Lichen-forming (foliose)	*Hypogymnia tubulosa*	0/8	0/8	3/10	0/10	A/S
Lichen-forming (foliose)	*Parmelia sulcata*	4/8	0/8	0/10	0/10	A/S
Lichen-forming (crustose)	*Placynthiella icmalea*/*dasaea*	2/8	0/8	0/10	0/10	A only
Lichen-forming (crustose)	*Placynthiella uliginosa*	1/8	0/8	0/10	0/10	S only
Lichen-forming (crustose)	*Lepraria neglecta*/*caesioalba*	0/8	0/8	0/10	1/10	S only
Lichenicolous/lichen parasite	*Epithamnolia* cf*. xanthoriae*	0/8	1/8	0/10	0/10	S only
Lichenicolous/lichen parasite	*Tremella macrobasidiata*	3/8	0/8	0/10	0/10	A/S
Dermatophyte	*Cutaneotrichosporon* cf*. mucoides*	1/8	0/8	0/10	0/10	A/S
Entomopathogen	*Cordyceps militaris s.l.*	0/8	0/8	1/10	0/10	A/S
Mycoparasite	*Eleutheromyces subulatus*	1/8	2/8	0/10	0/10	A/S
Mycoparasite (on boletes)	*Hypomyces chrysospermus*	0/8	1/8	0/10	0/10	A/S
Nectar/tap saprotroph (yeast)	*Candida zeylanoides*	1/8	1/8	1/10	1/10	S only
Nectar/tap saprotroph (yeast)	*Pichia kudriavzevii*	0/8	1/8	2/10	1/10	A/S
Nectar/tap saprotroph (yeast)	*Pichia fermentas*	0/8	1/8	2/10	1/10	A/S
Nectar/tap saprotroph (yeast)	*Pichia kluyveri*	0/8	0/8	2/10	1/10	A/S
Sooty mould	*Aureobasidium* cf*. pullulans*	7/8	3/8	7/10	8/10	A/S
Unclear, seems plant associated	*Chaetothyriales* 1 SH0888610.09FU	6/8	1/8	2/10	2/10	A only
Unclear, seems soil associated	*Capnodiales* 1 SH1035457.09FU	4/8	0/8	5/10	3/10	A/S
Unclear, seems soil or lichen associated	*Dothideomycetes* 3 SH0943484.09FU	4/8	0/8	3/10	3/10	A/S
Unclear, known from *Pinus sylvestris* leaf	*Helotiales* 1 SH1041195.10FU	0/8	0/8	4/10	6/10	A/S
Unclear, seems soil or plant associated	*Pleosporales* 1 SH0896666.09FU	3/8	3/8	1/10	1/10	A/S
Bryophyte	*Pleurozium schreberi*	1/8	0/8	0/10	1/10	NA
Vascular plant (tree)	*Betula pendula*/*pubescens*	2/8	1/8	0/10	6/10	NA
Vascular plant (grass)	*Agrostis* sp.	1/8	0/8	0/10	0/10	NA
Vascular plant (crop legume)	*Glycine max*	0/8	0/8	0/10	1/10	NA

**Fig. 1.**
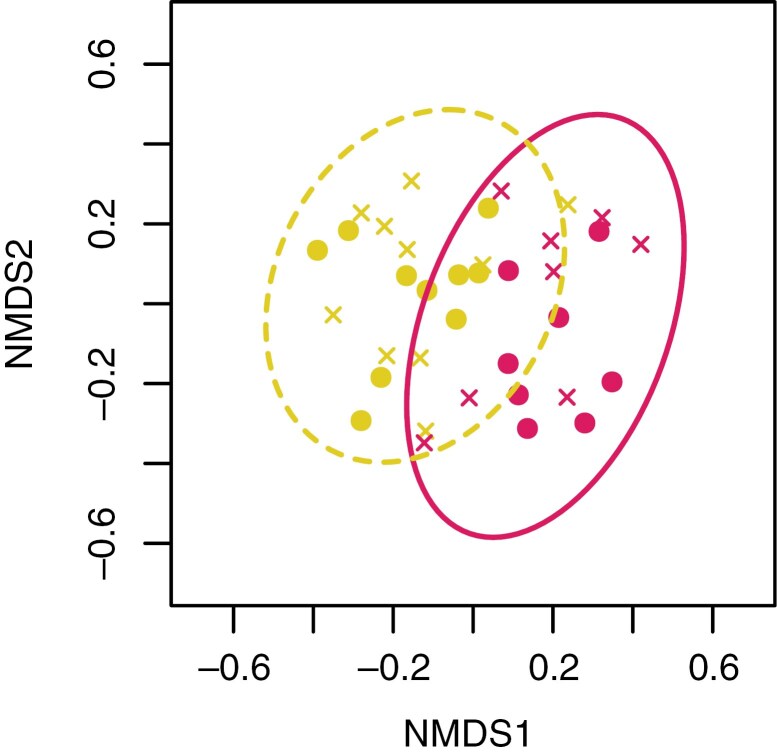
Representation of fungal operational taxonomic unit (OTU) assemblages in non-metric multidimensional scaling (NMDS) ordination space in robin (red) and goldcrest (gold) feathers (solid circles) and feet (crosses) (stress = 0.182, *k* = 3, *n* = 36). Ellipses represent 95 % confidence limits for group centroids, solid lines for robins and dashed lines for goldcrests. Other axis combinations and a comparison with environmental control samples can be found in Supplementary Data Figs S1 and S2.

Fungal assemblages from the two bird species are separated into distinct clusters in the NDMS ordination space (Fig. 1; Supplementary Data Fig. S1), and the environmental samples with fungi cluster clearly away from bird samples (Supplementary Data Fig. S2). The sampling position (feathers vs. feet) does not explain the patterns in the ordination. The bird species effect is statistically significant based on a PERMANOVA (*F* = 3.17, *P* = 0.001). This result is complicated by the robin samples having slightly but significantly higher multivariate dispersion than goldcrest samples (mean distance to group spatial mean 0.575 vs. 0.536; *F* = 5.25, *P* = 0.028).

**Fig. 2.**
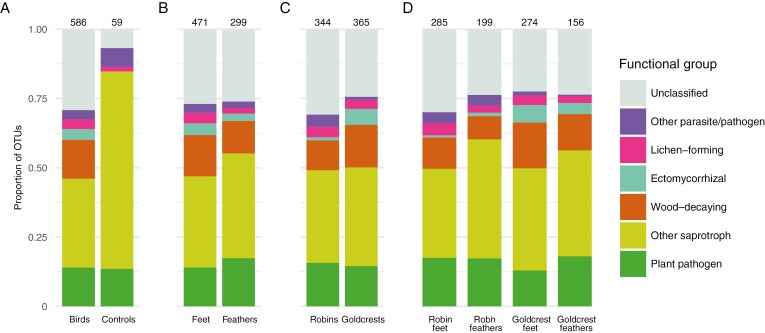
Proportion of taxa belonging to different functional lifestyles in the studied fungal assemblages, highlighting differences between bird samples and environmental controls (A), bird feet and feathers (B), robins and goldcrests (C), and feet and feathers by bird species (D). The total number of fungal operational taxonomic units for each sample type is shown above the bar.

**Fig. 3.**
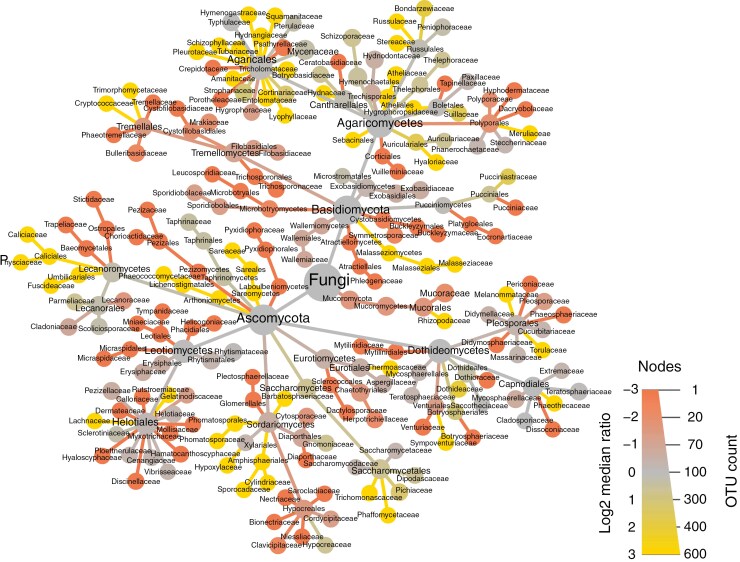
Taxonomic comparison of fungal assemblages in robins and goldcrests. The network tree illustrates all detected operational taxonomic units (OTUs) in a taxonomic hierarchy, starting from kingdom level (node Fungi in the centre) and ending on the family level (outermost nodes). The size of the node reflects the number of OTUs belonging to the corresponding taxon. The colour refers to the log_2_ median difference value calculated for each taxon, which is based on the difference in the proportion of samples from the two different bird species where that taxon was detected. Taxa with equal presence in both bird species are given log_2_ median difference values near 0 and are marked in grey. Taxa detected more often in goldcrests are given positive values (golden colour) and those in robins are given negative values (red colour).

The total number of OTUs was higher in goldcrests compared to robins (Fig. 2). Additionally, there were differences in the proportions of certain fungal taxonomic and/or functional lifestyles between the bird species (Figs 2 and 3). For instance, most ectomycorrhizal fungi were detected in goldcrests (21 OTUs) while only four such OTUs were detected in robins. Other pathogens and parasites, including myco- and entomopathogens, were more common in robins (24 vs. 14 OTUs in goldcrests, Fig. 2). Most representatives of Agaricales were more frequent in goldcrests, whereas many taxa in Helotiales and Hypocreales were characteristic for robins (Fig. 3).

**Fig. 4.**
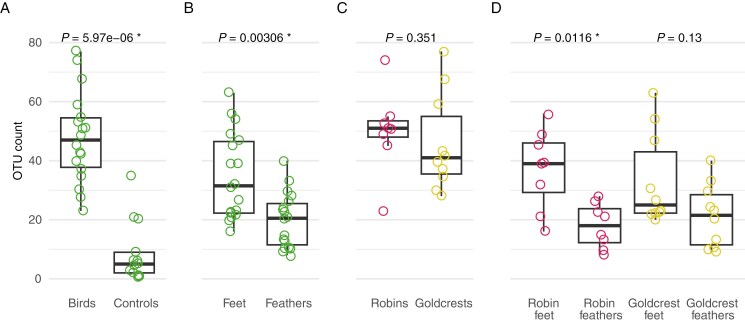
Number of fungal operational taxonomic units (OTUs) across different sample types: between bird samples and environmental controls (A), bird feet and feathers (B), robins and goldcrests (C), and feet and feathers by bird species (D). Data points from robins are in red and from goldcrests in gold. Results of statistical comparisons between sample types using the non-parametric Mann–Whitney U test are shown overlaid on the boxplot, and significant *P*-values are marked with an asterisk. In D, the statistical comparisons are between sampling position within the same bird species.

The number of fungal OTUs detected from individual birds ranged from 23 to 77 (Fig. 4A). While goldcrests had a higher total number of taxa, on average individual goldcrests harboured fewer taxa than individual robins (Fig. 4B). Feather samples had fewer OTUs than foot samples in total and on average, and this was the case also when analysing both bird species separately (Figs 2 and 4C, D). None of the major taxonomic functional groups were more frequently present in feathers than in feet.

**Fig. 5.**
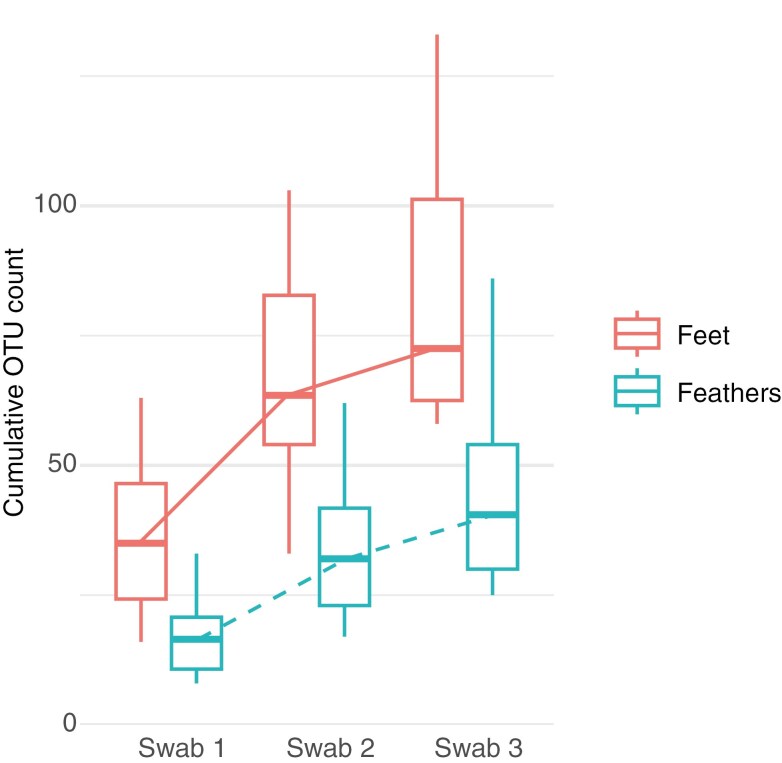
Cumulative fungal operational taxonomic unit (OTU) count for three consecutive replicate sampling swabs of the same individual bird (*n* = 3 for both goldcrests and robins). Lines connect median OTU counts across the consecutive swabs. Red boxes and solid lines represent samples from bird feet, blue boxes and dashed lines from bird feathers.

In bird individuals that were sampled using three consecutive swabs, the number of detected OTUs increased with each consecutive sample, but the overall trend shows some saturation (Fig. 5). This confirms that the fungal assemblages detected with a single swab represent only a subset of the total pool of taxa attached to each bird.

Of the bird-derived fungal OTUs, 63 and 74 % were detected in global large-scale eDNA sampling campaigns for aerial fungal eDNA (Ovaskainen *et al.*, 2024) and soil fungal eDNA (Tedersoo *et al.*, 2021), respectively. Of the OTUs detected in birds, 96 (or 16 % of all detections) were not present in either of the comparison datasets.

Most of the non-target, non-fungal taxa detected represent green algae (27 OTUs), both from lichen-symbiotic (e.g. *Trebouxia*, *Asterochloris*) and non-symbiotic (e.g. *Apatococcus*) genera. Detected vascular plants include *Betula pendula/pubescens* and *Agrostis* sp., both very common in the area. Other vascular plants include agricultural species such as *Glycine max*, which may represent plant remains attached to birds during granivory or otherwise physical contact with plant material. The pleurocarpous, very common forest floor moss *Pleurozium schreberi* is also detected. Other taxa include the common forest spider *Linyphia trianglularis*, a few collembolans (e.g. *Allacma fusca* and *Sminthurus viridis*) and the planktonic ciliate *Helicostomella subulata*.

## DISCUSSION

The considerable diversity of fungi we detected shows that birds can transport a wide variety of taxa. Our results also indicate that animal vectors may be involved in the dispersal ecology of a plethora of fungal species that have not been previously considered as being animal-transported. For most taxa, our results provide the first cue of such an interaction with a bird.

Fungal DNA detected on bird feathers and feet may originate from many types of fungal sources, including sexual or asexual spores, hyphae, vegetative propagules and thallus fragments, or fragments of soil, biocrust or decaying organic material containing fungal biomass. These may have attached to birds via direct contact with fungal fruiting bodies, or indirectly as airborne or previously deposited fungal material. For many soil fungi, especially those without spore-producing structures, the DNA has probably attached to birds within soil or dust particles. Bird-mediated transport of such taxa should thus be considered in the framework of community coalescence, or transport of entire (microbial) communities (Rillig *et al.*, 2015; Castledine *et al.*, 2020).

The detected fungal OTUs can be roughly divided into three groups: (1) substrate-specific fungi (picked up by direct or secondary contact in a manner that may be directly relevant for fungal dispersal), (2) ubiquitous environmental fungi (presumably picked up by density-dependent, sporadic secondary contact that does not have direct relevance for fungal dispersal) and (3) bird-resident fungi (fungal taxa specialized to live on birds or their remains).

Most detected fungal taxa live on specific substrates or in particular habitats, suggesting that the transport service of the bird might be relevant for their dispersal ecology. In particular, cases where the substrate requirements of a fungus are strict and match the habitat preferences of the bird may be indicative of passive directed dispersal (da Silva *et al.*, 2016; Mason *et al.*, 2022). Directed dispersal is considered high-quality dispersal with a high probability of successful establishment, since the vector selectively transports the propagules to preferred habitats or substrates (Schupp *et al.*, 2010). This gives a greater chance of effective dispersal occurring when compared to a more random movement path in, for example, wind dispersal. Propagules picked up by secondary contact can be expected to have lower viability as they have already been transported by other vectors, such as wind.

The fungi detected from birds include at least 92 taxa of plant pathogenic fungi, such as leaf/canopy pathogens and wood-decaying fungi, some of these specific to either conifers, deciduous trees, graminoids or forbs. A few detected fungi are parasitic on insects (e.g. *Cordyceps militaris s.l.*), parasitic on other fungi (e.g. *Hypomyces chrysospermus*) or are associated with lichens (e.g. *Tremella macrobasidiata*). Some of the plant pathogens are economically important pests, such as *Heterobasidium annosum* and *H. parviporum*, which cause heart rot of *Picea abies* and *Pinus sylvestris*, respectively, and *Lophodermium* species which induce needle cast of conifers. For foliar pathogens, the probable pickup route is via direct contact with propagule-producing structures in the canopy or on the forest floor, or secondary deposition from air or surfaces, especially during mass sporulation events of these fungi. *Heterobasidion* species typically produce resupinate fruiting bodies and small spores in basal crevices of conifers (Niemelä, 2005) and are thought to primarily infect new host trees via relatively short-distance aerial dispersal to stumps or wounds (Garbelotto and Gonthier, 2013). The most probable origin of *Heterobasidion* DNA, which was detected especially from goldcrest feet, is secondary contact with spores from the air or surfaces, as the primarily canopy-dwelling goldcrests are unlikely to come into direct contact with the concealed fruiting bodies. While birds are thus unlikely to play a central role in the local dispersal dynamics of these fungi, they might still facilitate occasional events of long-distance dispersal (Gonthier *et al*., 2001; Garbelotto and Gonthier, 2013). This could be especially true for migrating goldcrests, which visit conifer-dominated forests suitable both for goldcrest foraging and *Heterobasidion* infection.

Systemic plant parasites of the genus *Exobasidium* spp. were surprisingly common, especially in robin feet. Many of these fungi typically form colourful or inconspicuous leaf spots or galls in species of the Ericaceae, including the common and often dominant berry-bearing dwarf shrubs *Vaccinium myrtillus* and *V. vitis-idaea* (Stenroos *et al.*, 2020). As robins are opportunistically frugivorous (Debussche and Isenmann, 1985; Honkavaara *et al.*, 2007), the uptake of *Exobasidium* spores presumably occurs via direct contact with foraging birds and may greatly facilitate the dispersal of these fungal parasites. This type of interaction can be expected to be even stronger in bird species such as thrushes (*Turdus* spp.), which are more often frugivorous and typically track fruit crop resources (Tellería *et al.*, 2014). It is generally known that the colourful appearance of the spore-producing galls of many pathogenic fungi can function as ‘pseudo-flowers’, visual deceptive lures attracting pollinating insects, which then act as fungal dispersal vectors (Ngugi and Scherm, 2006). Evidence from vector exclusion experiments suggests that arthropods can contribute to *Exobasidium* dispersal (Newell *et al.*, 2023), but these experimental setups have also excluded larger potential dispersal vectors, such as birds. If the sporulating structures induced by some *Exobasidium* indeed mimic fruit, frugivorous birds might be very important in their dispersal dynamics, even on large geographical scales. Similar hypotheses about visual cues have been suggested for conspicuous colours and morphologies present in some seemingly bird-dispersed endemic truffles of New Zealand (Beever and Lebel, 2014).

The agaricoid genus *Mycena* is very common in our dataset, represented by more than 20 species, many of them found in goldcrests and especially their feet. The small fruiting bodies of these decomposing fungi are typically associated with litter, soil or woody debris on the ground (Aronsen and Læssøe, 2016). Thus, their common presence in the feet of primarily canopy-dwelling goldcrests is unexpected. Seasonal mass sporulation events, where spores of these fungi are sporadically released into the environment in massive quantities, could explain their high prevalence. Bird mycophagy, classically considered rare, but recently suggested to be more common than previously thought (Elliott *et al.*, 2019), seems unlikely for primarily insectivorous goldcrests during autumn when insect prey is readily available.

Ectomycorrhizal fungi are comparatively rare in our data, but characteristically found especially in goldcrest feet. This group includes common agaricoid, boletoid, corticioid and clavarioid species typical for conifer-dominated heath forests. Additionally, many mushroom-forming wood-decaying fungi detected in goldcrests have similar habitat preferences. This suggests matching of habitat preferences of the bird vector and the transported fungi. While direct pickup of fungal propagules (probably spores) is possible, secondary pickup seems more likely, especially for goldcrests. If this is the case, the relative importance of potential bird-mediated dispersal appears low in comparison to aerial dispersal, at least over short distances.

For longer transport distances, such as between forest patches in a fragmented landscape, birds could be important dispersal vectors when compared to wind dispersal. This is because aerial spore occurrence is known to drop quite sharply at forest edges (Abrego *et al.*, 2020) and spore collection experiments from forest floor fungi typically show dispersal kernels where most spores do not reach the upper atmosphere but are deposited near the spore source (Nordén and Larsson, 2000; Galante *et al.*, 2011; Norros *et al.*, 2012; Peay *et al.*, 2012). Habitat-directed bird movement could thus transport propagules to new forest patches suitable for establishment beyond typical short-range aerial dispersal distances.

Potential transport distances of mobile and migratory birds range from the landscape to the intercontinental scale. Based on ringing data, the maximal migration distances in 24 h estimated for Finnish goldcrests is 646 km and for robins 519 km (Valkama *et al.*, 2014). The turnover time of propagules attached to birds is poorly known, but experiments suggest that fungal spores can stay viable attached to bird feathers for at least 45 d (Warner and French, 1970). Taken together, this indicates that both bird species have the potential to transport fungal propagules even on the intercontinental scale.

Most of the lichen-forming fungi detected from the birds are common epiphytic macrolichens. Among these, *Parmelia sulcata* and *Hypogymnia tubulosa* are detected exclusively in robins and goldcrests, respectively. *Parmelia sulcata* typically grows on the bark of deciduous trees and *H. tubulosa* on conifer bark and branches. These match the preferred habitats of their respective bird vectors, indicating the possibility for passive directed transport of the lichen species to suitable substrates by the two bird species.

Most lichen species detected are primarily sorediate, i.e. they produce large amounts of relatively large (20–50 μm) vegetative symbiotic propagules (soredia), which allow the co-dispersal of all lichen symbiotic partners (Nash, 2008). Typically, such lichens produce sexual spores only rarely. In previous work soredia have been observed directly attached to birds (Johansson *et al.*, 2021), and the detection of lichen photobiont eDNA in our data corroborates the presence of symbiotic propagules. As soredia are larger than typical ascospores, they seem less suited for wind dispersal (Ronnas *et al*., 2017; but see Marshall, 1996; Tormo *et al.*, 2001) and are probably picked up by the birds via direct contact with the sorediate thalli. Generally, bird-mediated transport of soredia can be expected to result in longer transport distances than when carried by wind. Additionally, large symbiotic propagules have high establishment probability and high viability (Černajová and Škaloud, 2020), suggesting that bird-mediated soredial dispersal may be of high quality both via spatial directedness and via propagule properties.

Species of the crustose lichen genus *Scoliciosporum* are among the most common fungal taxa detected on the two birds, especially on their feet. Species-level OTU annotation is difficult in *Scoliciosporum* due to taxonomic uncertainties and lack of reference sequences, but the most likely detected species include *S. umbrinum*, *S. chlorococcum* and allied species, all with minute, poorly developed non-corticated thalli. A high occurrence of *Scoliciosporum* species was documented in lichen-targeted eDNA survey on beech bark swabs (Dreyling *et al.,* 2024), and representatives of the genus have also been common in aerial fungal eDNA surveys (Ovaskainen *et al.*, 2024). These findings indicate that species of *Scoliciosporum* are very common in the landscape but difficult to visually confirm. They also highlight that many minute crustose lichens are probably severely underdetected and under-recorded in traditional biodiversity surveys.

About 200 fungal OTUs can only be identified to a high taxonomic level. Most of these are small ascomycetes belonging to the species-rich orders Pleosporales, Helotiales and Capnodiales and the class Dothideomycetes. While the Linnean name of many of these taxa remains unclear, their SHs in the UNITE database are explicit and can allow some ecological inference. For example, OTU Helotiales 1 (SH1041195.10FU) is reasonably common in goldcrests – especially in feathers – but absent from robins. A sequence belonging to this SH has previously been detected in Finland from *Pinus sylvestris* needles using culture approaches (Terhonen *et al.*, 2011), suggesting an ecological link to *Pinus* forests, which well matches the typical habitat of goldcrests.

Our dataset includes many fungal taxa with unspecified ecologies which are commonly encountered in eDNA surveys. Many such taxa are common in the dataset and represent filamentous moulds, unspecific saprotrophs and yeasts (e.g. in the genera *Cladosporium*, *Aspergillus*, *Mucor*, *Pichia* and *Candida*). Many of these fungi were also present in the environmental control samples, are known to reproduce massively via conidia and have been detected in previous work on birds (Alfonzo *et al.*, 2013; Francesca *et al.*, 2014). Such fungi can be almost omnipresent in the ambient environment and are probably easily picked up by birds via secondary contact. Some detected fungi are dermatophytes (e.g. *Cutaneotrichon*) and may represent pathogens or commensalist residents on the bird, while others may even be opportunistic human pathogens. The potential pathogens found in this study may be of environmental origin and not acutely pathogenic on the sampled bird, but birds may play a role in their transmission to new hosts or locations. Their interaction dynamics with the vector birds might be best approached in terms of disease dynamics.

## CONCLUDING REMARKS

Like many interactions between species, the dispersal interactions discussed here are expected to be sensitive to environmental change. Alarming results from defaunation studies show that the erosion of seed dispersal interaction networks can have major ecological and evolutionary consequences (Galetti *et al.*, 2013; Bello *et al.*, 2015; Valiente-Banuet *et al.*, 2015), and such interactions can be under threat and of high conservation concern (Mendes *et al*., 2024). It has been estimated that the abundance of several bird species in Finland has dropped markedly in the last 50 years, accompanied by community composition change, for various anthropogenic reasons (Lehikoinen *et al.*, 2019; Lehikoinen and Väisänen, 2023). These changes are likely to alter and even disrupt also bird–fungal dispersal interactions, potentially affecting fungal populations, communities and biodiversity–ecosystem function relationships in fungi (Runnel *et al.*, 2024). A holistic understanding of fungal dispersal requires not only combining theoretical and empirical approaches, but also to understand the biology of potential animal dispersal vectors.

## SUPPLEMENTARY DATA

Supplementary data are available at *Annals of Botany* online and consist of the following.

Table S1. Full dataset of detected OTUs, their taxon assignments and the presence–absence OTU table across samples, including sample metadata. Fig. S1. Expanded visualization of the fungal OTU assemblages in NMDS ordination space (Fig. 1) with all pairwise NDMS axis combinations. Fig. S2. Expanded visualization of the fungal OTU assemblages, including environmental controls, in NMDS ordination space (Fig. 1) with all pairwise NDMS axis combinations.

mcaf077_suppl_Supplementary_Table_S1

mcaf077_suppl_Supplementary_Figures_S1-S2
