# Supplementary material for: Diversity of fungi attached to birds corresponds to the habitat ecologies of their avian dispersal vectors
Source: Ann Bot. 2025 May 3;136(4):721–32. doi: 10.1093/aob/mcaf077 (PMC12464955; doi:10.1093/aob/mcaf077)
Supplement: mcaf077_suppl_Supplementary_Figures_S1-S2 [file mcaf077_suppl_supplementary_figures_s1-s2.docx]

Supplementary Figure 1. Representation of fungal operational taxonomical unit (OTU) assemblages in non-metric multidimensional scaling (NMDS) ordination space in robin (red) and goldcrest (gold) feathers (solid circles) and feet (crosses) (stress = 0.182, k = 3, n = 36). Ellipses represent 95% confidence limits for group centroids, solid lines for robins and dashed lines for goldcrests. The three ordinations represent all pairwise combinations of the NDMS axes.

Supplementary Figure 2. Representation of fungal operational taxonomical unit (OTU) assemblages in non-metric multidimensional scaling (NMDS) ordination space in robin (red) and goldcrest (gold) feathers (solid circles) and feet (crosses) and environmental controls (blue triangles) (stress = 0.171, k = 3, n = 44). Ellipses represent 95% confidence limits for group centroids, solid lines for environmental controls, dashed lines for robins and dotted lines for goldcrests. The three ordinations represent all pairwise combinations of the NDMS axes. Environmental control samples which included less than three fungal OTUs were excluded from the NMDS, as almost empty assemblages they render the ordination meaningless for the focal samples.
